# High prevalence of human cytomegalovirus in carotid atherosclerotic plaques obtained from Russian patients undergoing carotid endarterectomy

**DOI:** 10.1186/2042-4280-4-3

**Published:** 2013-11-14

**Authors:** Koon-Chu Yaiw, Olga Ovchinnikova, Chato Taher, Abdul-Aleem Mohammad, Belghis Davoudi, Eugene Shlyakhto, Oxana Rotar, Alexandra Konradi, Vanessa Wilhelmi, Afsar Rahbar, Lynn Butler, Alice Assinger, Cecilia Söderberg-Nauclér

**Affiliations:** 1 Department of Medicine, Center for Molecular Medicine, CMM L8:03, Karolinska Institutet, Solna, Stockholm SE-171 76, Sweden; 2 Almazov Federal Center for Heart, Blood and Endocrinology, St. Petersburg, Russia

**Keywords:** Cytomegalovirus, Serostatus, Carotid atherosclerotic plaque, Viral antigens in plaques, Inflammatory markers

## Abstract

**Background:**

Human cytomegalovirus (HCMV) infection is associated with cardiovascular disease (CVD) but the role of this virus in CVD progression remains unclear. We aimed to examine the HCMV serostatus in Russian patients (n = 90) who had undergone carotid endarterectomy (CEA) and controls (n = 82) as well as to determine the prevalence of HCMV immediate early (IE) and late (LA) antigens in carotid atherosclerotic plaques obtained from 89 patients. In addition, we sought to determine whether HCMV infection was associated with inflammatory activity in the plaque by quantifying infiltrating CD3 and CD68 positive cells and 5-LO immunoreactivity.

**Methods:**

HCMV serology was assessed with ELISA and immunohistochemistry staining was performed to detect HCMV antigens, CD3, CD68 and 5-LO reactivity. The Fisher’s exact test was used to compare i) seroprevalence of HCMV IgG between patients and controls and ii) HCMV-positive or –negative to that of CD3, CD68 and 5-LO immunoreactive cells in plaque samples. The student-*t* test was performed to connote the significance level of mean optical density between patients and controls.

**Results:**

The seroprevalence for HCMV IgG was high in both patients and controls (99% and 98%, respectively). Controls had significantly higher IgG titers for HCMV compared with patients (p = 0.0148). Strikingly, we found a high prevalence of HCMV antigens in atherosclerotic plaques; 57/89 (64%) and 47/87 (54%) were HCMV IE and LA positive, respectively. Most plaques had rather low HCMV reactivity with distinct areas of HCMV-positive cells mainly detected in shoulder regions of the plaques, but also in the area adjacent to the necrotic core and fibrous cap. In plaques, the cellular targets for HCMV infection appeared to be mainly macrophages/foam cells and smooth muscle cells. HCMV-positive plaques trended to be associated with increased numbers of CD68 positive macrophages and CD3 positive T cells, while 5-LO reactivity was high in both HCMV-positive and HCMV-negative plaques.

**Conclusions:**

In Russian patients undergoing CEA, HCMV proteins are abundantly expressed in carotid plaques and may contribute to the inflammatory response in plaques via enhanced infiltration of CD68 and CD3 cells.

## Background

Human cytomegalovirus (HCMV) is a DNA virus that belongs to the family *Herpesviridae*[1]. It is a ubiquitous but species-specific virus that remains latent in host cells upon primary infection. Its seroprevalence varies from 40-100% depending on age, socioeconomic status and geographical locations [2]. HCMV is known as an opportunistic infection in immunocompromised individuals and it is the main etiological agent responsible for congenital viral diseases [1]. Accumulating evidence also supports a plausible role of microbial infection in the pathogenesis of cardiovascular disease (CVD) [3]. For example, many studies show the presence and probable active role of microbes such as *Chlamydia pneumonia*, *Helicobacter pylori* and certain periodontal pathogens or viruses, such as herpes simplex virus and HCMV in the pathogenesis of CVD [3,4]. Among these, HCMV and *Chlamydia pneumonia* provide the strongest evidence of a link to CVD through seroepidemiological, clinical and experimental models [5-7].

HCMV has been proposed to influence various cardiovascular disease processes [3,8,9]. HCMV nucleic acids and/or antigens have been detected in atherosclerotic plaques [10,11]. Furthermore, elevated antibody levels against HCMV are positively correlated with atherosclerosis [12], coronary artery disease [13] and more recently, with CVD mortality [5,14-16]. In a mouse model, Cheng et al. provided evidence that murine CMV possibly results in enhanced arterial blood pressure through increased angiotensin II [17]. Interestingly, patients with essential hypertension have higher HCMV-specific microRNA levels in plasma [18]. However, some studies have also failed to detect the presence of HCMV in atherosclerotic plaques or show any association between this virus and CVD [19,20]. Hence, the role of HCMV in CVD remains controversial.

Inflammation is a well-established atherogenesis promoter that increases CVD risk and contributes to plaque rupture [21,22]. Among known proinflammatory mediators in CVD, leukotrienes are potent chemotactic molecules, which attract leukocytes and induce vascular permeability and smooth muscle cell contraction. 5-lipoxygenase (5-LO) catalyzes the first steps in the conversion of arachidonic acid to leukotrienes, and is implied in the pathogenesis of atherosclerosis, restenosis and plaque instability [23]. In atherosclerotic plaques, the expression of 5-LO is mainly restricted to granulocytes, monocytes/macrophages, foam cells, dendritic cells, B cells and mast cells but not T cells [24]. HCMV induces expression of both 5-LO mRNA and proteins as well as leukotriene B4 (LTB4) production in infected vascular smooth muscle cells [25], which otherwise are unable to produce LTB4. HCMV could thereby contribute to local inflammation in the vascular wall and drive CVD progression. Inflammation is also a driving force for reactivation of latent HCMV [26,27], and replication of this virus in macrophages [28]. We therefore hypothesize that HCMV positivity is associated with inflammation. In this study, we aimed to determine the HCMV serostatus in Russian patients who underwent carotid endarterectomy (CEA) and in controls. We also sought to examine the presence of HCMV immediate early (IE) and late (LA) antigens in carotid atherosclerotic plaques and to search for an association between HCMV positivity and enhanced presence of inflammatory markers, i.e.: 5-LO, CD3 and CD68 in CEA biopsy specimens.

## Materials and methods

### Specimens

Both sera (n = 90) and human atherosclerotic plaque tissue samples (n = 89) were obtained from Russian patients with CEA that were stored in St. Petersburg Investigation of Carotid Endarterectomies (SPICE) Biobank (Table 1). The Ethical Committee of Palvov State Medical University of St. Petersburg approved the studies. Pieces of plaques were embedded in Optimal Cutting Temperature compound (Sakura Finetek, the Netherlands) and frozen for immunohistochemistry or nucleic acid analysis. The control sera were from 83 control individuals and the study was approved by the Institutional Review Board on cardiology and endocrinology from the Almazov Federal Heart, Blood and Endocrinology Centre, St. Petersburg. Among these controls, 67 of them had ≤ 1.2 mm carotid intima-media thickness and 16 of them had low degree of atherosclerosis whom at present had no symptoms.

**Table 1:** Patient demographics and characteristics

**Clinical parameters**	**Values**
Male : Female (n)	72: 18
Age (mean ± SD)	62.0 ± 8.2
Symptoms, yes (%)	38.6 (32 of n*83)
Ipsilateral carotid stenosis (%)	78.0 ± 10.3 (of n*77)
**Risk factors:**	
Smoking, yes (%)	48.1 (37 of n*77)
BMI (kg/m^2^)	26.7 ± 4.7 (of n*78)
Diabetes mellitus type 2, yes (%)	26.3 (21 of n*80)
Hypertension, yes (%)	96.3 (78 of n*81)
Total cholesterol (mmol/L)	5.6 ± 1.3 (of n*84)
**Treatment:**	
Statins (%)	33.3% (27 of n*81)
Antiplatelet drugs (%)	82.5 (66 of n*80)
Antihypertensive drugs (%)	73.8 (59 of n*80)

*BMI* body mass index, values are mean ± SD if not indicated otherwise, n* number of patients with available variant; Symptoms refer to transient ischemic attack, ischemic stroke or retinal ischemia.

### Enzyme-linked immunosorbent assay (ELISA)

HCMV IgM titers were determined by ELISA (Enzygnost anti-CMV/IgM, Siemens, Germany) and IgG by an in-house anti-CMV/IgG ELISA assay, Karolinska University Hospital, Sweden). This assay is used for routine clinical diagnostics at the accredited Clinical Virology Laboratory at the Karolinska University Hospital. We obtained comparable results by using this method and two commercial kits (Afsar Rahbar, personal communication). The anti-CMV/IgM ELISA was assayed in duplicates according to the manual’s instruction. For the anti-CMV/IgG ELISA, an in-house prepared 96-well plate coated with CMV/control antigens was washed 3 times with washing buffer (0.9% NaCl/0.05% Tween 20), incubated at 37°C, 60 mins with plasma (1:500), thereafter washed 4 times and incubated with polyclonal rabbit anti-human IgG conjugated to horseradish peroxidase (Dako, Denmark), 1:5000 in 1% BSA/PBS for 5 mins on a shaker at RT and thereafter for 60 mins at 37°C. After 4 washings, reactivity was detected using o-phenylenediamine. The reaction was stopped with 2.5 M sulphuric acid (Merck, Germany). The O.D. was read at 492 nm. The cut-off value for positivity was an O.D. > 0.2.

### Immunohistochemistry (IHC)

IHC was performed with 8–10 μm thick acetone/methanol-fixed cryosections using ImmPRESS detection system. After endogenous peroxidase quenching, sections were sequentially incubated with protein block, Fc receptor blocker, followed by normal horse serum, all for 20 mins at RT. Primary antibodies, mouse anti-HCMV immediate early (IE, Millipore, MAB810R, clone 8B1.2, 1:500), mouse anti-HCMV late proteins (Millipore, MAB8127, clone 1G5.2, 1:500), mouse anti-human CD68 (Dako, M0876, clone PG-M1,1:200), mouse anti-human CD3 (Dako, M7254, clone F7.2.38, 1:100) and rabbit anti-5-LO (Abcam, ab39347, 1:400) were applied and incubated overnight at 4°C. Negative controls were prepared by omitting the primary antibody (TBS only) or with isotype controls. HCMV VR1814-infected endothelial cells served as positive controls. Immunoreactivity was detected with diaminobenzidine (DAB) and counterstained with hematoxylin before microscope viewing. The staining was graded according to the estimated percentage of positive cells with grade I denoting <25%; grade II, 25-50%; grade III, 51-75% and grade IV, >75% as previously described [29].

### TaqMan PCR

Tissue plaques samples were cut into small pieces, incubated with Proteinase K overnight and DNA was extracted using DNeasy Blood & Tissue kit (Qiagen). The primers and probe sequences used for the HCMV IE DNA detection were as previously described [30]. The TaqMan reaction was performed with fast master mix using ABI Prism 7900HT with the default thermocycling profile using 50 ng as template in triplicates along with positive and non-template controls. We used HCMV-infected fibroblast cells as a positive control and a non-template control of water as a negative control. In addition, we also utilized the first WHO International Standard for HCMV developed by NIBSC (code 09/162) to make a standard curve in order to calculate the viral copy number. In brief, the assigned 5 × 10^6^ IU of HCMV in 1 ml of nuclease-free water was subjected to DNA extraction using QIAamp MinElute Virus Spin Kits (Qiagen) and the DNA was eluted with 100 μl of nuclease-free water. A standard curve was then generated using a serial of ten-fold dilution with two microliter of each dilution was used in a total volume of 10 μl reaction.

### Statistical analysis

All statistical analysis was performed using GraphPad Prism 5 software. The Fisher’s exact test was used to compare i) seroprevalence of HCMV IgG between patients and controls and ii) HCMV-positive or –negative to that of CD3, CD68 and 5-LO immunoreactive cells in plaque samples. The student-*t* test was performed to connote the significance level of mean optical density between patients and controls. A p value < 0.05 was considered statistically significant.

## Results

### HCMV IgG titers are lower among CEA patients than controls

**Figure 1:**
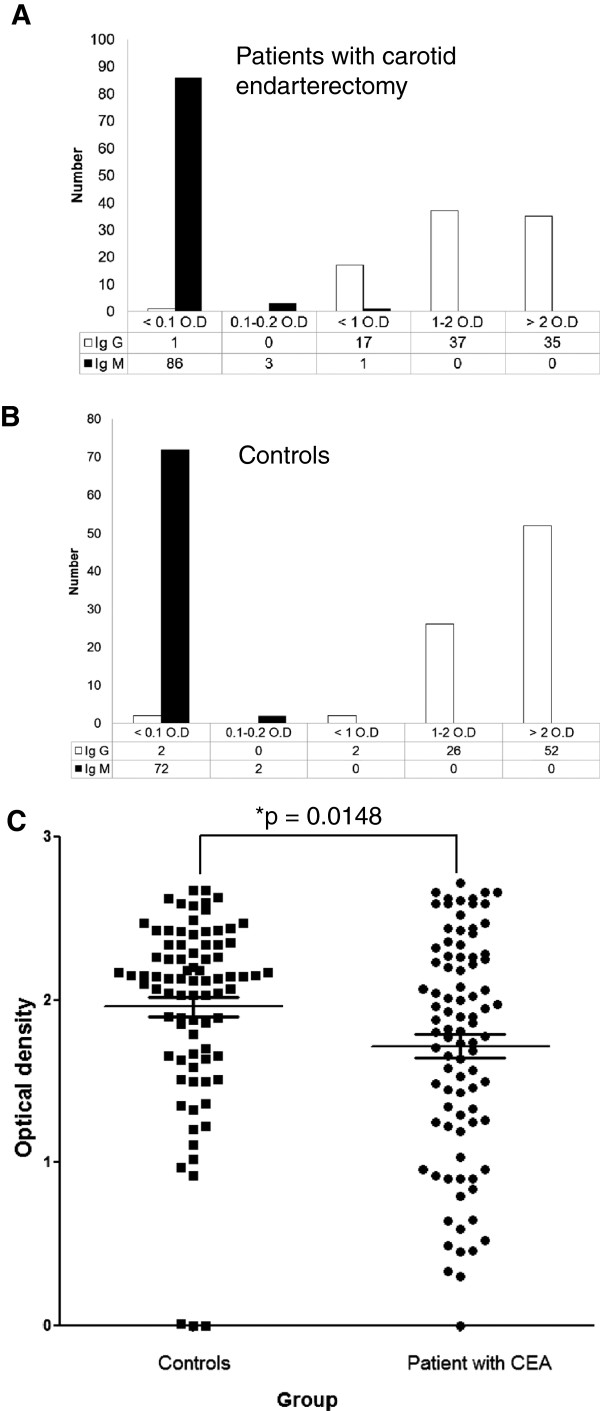
**Serological analysis of human cytomegalovirus (HCMV) antibodies in Russian patients with carotid endarterectomy (CEA) and controls. (A)** and **(B)** Bar graphs show seroprevalence of IgG and IgM antibodies against HCMV in patient with CEA or controls, respectively. **(C)** Scatter plot shows optical density of IgG between patients with CEA and controls. Carotid endarterectomy, CEA; Optical density, O.D.; *p < 0.05.

Table 1 shows all the available patient demographics and characteristics for 90 sera from CEA patients that were examined in our study. The ratio of males to females was 4:1 (i.e. males, n = 72 and females, n = 18) and the mean age was 62.0 ± 8.2 (mean ± SD) for males and 65.8 ± 8.6 for females. 89 of 90 (99%) sera from CEA patients were positive for HCMV IgG. One (1%) was positive for IgM and three (3%) were equivocal (Figure 1A). From controls, we obtained 83 serum samples; HCMV IgG and IgM titres were determined in 82 samples by ELISA (1 sample was insufficient in quantity for the assay). Females constituted the major gender (n = 56) among controls (ratio male to female approximately 1:2) with mean age of 56.8 ± 9.5 for females compared to 52.2 ± 14.5 for males. The controls showed similar patterns of HCMV IgG seroprevalence; 80/82 (98%) were HCMV IgG positive (Figure 1B), none was IgM positive and 2/74 (3%) were equivocal for IgM (Figure 1B). Thus, there was no significant difference in HCMV IgG positivity between patients with CEA and controls (p = 0.6059). However, we observed that controls had a significantly higher O.D. value for HCMV IgG compared to patients (1.95 ± 0.57 and 1.71 ± 0.68, respectively; p = 0.0148, Figure 1C). Removal of 16 controls with low degree of atherosclerosis resulted in the same conclusion either for HCMV IgG positivity or mean O.D. between patient with CEA and controls.

### HCMV IE and LA are frequently present in atherosclerotic plaques

**Figure 2:**
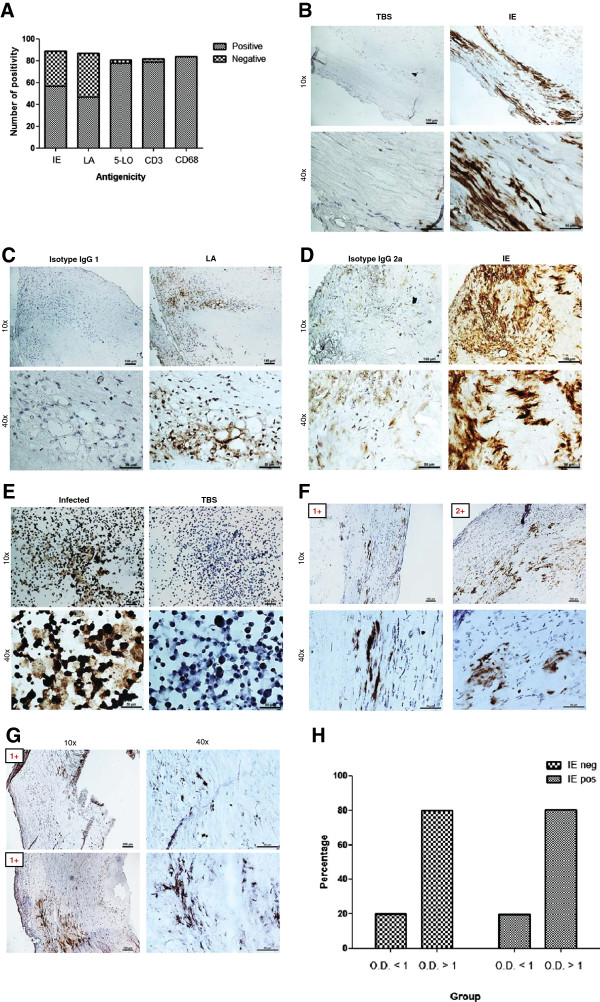
**Immunoreactivity and grading of human cytomegalovirus (HCMV) antigens and inflammatory markers in human carotid atherosclerotic plaques as assayed by immunohistochemistry (IHC) staining. (A)** Summary of IHC results on viral immediate early (IE), late (LA), 5-LO, CD3 and CD68 in plaques. **(B-E)** Panel of controls consist of omitting primary antibody (or Tris-buffered saline, TBS only) instead of IE **(B)**, isotype control for LA **(C)**, isotype control for IE **(D)**, and virus-infected endothelial cells **(E). (F-G)** Immunoreactivity and grading of IE **(F)** or LA **(G)** in plaques. **(H)** Association between HCMV IgG antibodies and its IE antigens burden. Positivity was revealed by diaminobenzidine (DAB), brown products; Optical density, O.D.

We examined 89 frozen tissue samples obtained from CEA patients for HCMV IE and 87 for HCMV LA. Strikingly, 57/89 (64%) and 47/87 (54%) were positive for HCMV IE and LA, respectively (Figure 2A). Minimal or no HCMV immunoreactivity was detected in sections incubated with TBS (i.e. omitting primary antibody) (Figure 2B) or using isotype control antibodies (IgG1 and IgG2a) (Figure 2C-2D, respectively). Endothelial cells infected *in vitro* were positive for HCMV IE and LA (Figure 2E).

To confirm the presence of HCMV nucleic acids in plaques, we randomly selected 9 samples from HCMV IHC-positive and 9 samples of IHC-negative tissue plaques, isolated DNA and performed TaqMan PCR analysis for HCMV. We detected 4/9 (44%) samples positive for IE DNA, while none of nine IHC-negative plaques was positive for HCMV IE (Table 2). We used HCMV-infected fibroblast cells as positive control for the PCR assay, which was always positive, with an average threshold cycle = 15.0 ± 0.2 (equivalent to 7.7 × 10^9^ IU/ml). The non-template control consisting of water was consistently negative. The sensitivity limit of our assay was determined to be 765 IU/ml using the WHO International Standard for HCMV. We failed to extract RNA from 10 samples (each 10 tissues section slices) that was sufficient in both quantity and quality to perform TaqMan PCR as assessed by NanoDrop 2000 (Thermo Scientific).

**Table 2:** TaqMan PCR analysis and cellular localization of viral antigens on selected IHC-positive or -negative for HCMV antigens

**Sample**	**Viral antigens localization as revealed by immunohistochemistry**	**TaqMan PCR (IU/ml)**
**CP 03**	Endothelial cells, macrophage/foam cells, smooth muscle cells	10,298
**CP 05**	Endothelial cells, macrophage/foam cells, smooth muscle cells	2,598
**CP 30**	Endothelial cells +/-, macrophage/foam cells, smooth muscle cells	Undetermined
**CP 31**	Smooth muscle cells	Undetermined
**CP 36**	Macrophage/foam cells, inflammatory cells, smooth muscle cells +/-	Undetermined
**CP 37**	Endothelial cells, macrophage/foam cells, smooth muscle cells	7,956
**CP 44**	Endothelial cells, macrophage/foam cells, inflammatory cells, smooth muscle cells	Undetermined
**CP 47**	Macrophage/foam cells, smooth muscle cells +/-	Undetermined
**CP 101**	Macrophage/foam cells, smooth muscle cells+/-	4,718
**CP 08**	None detected	Undetermined
**CP 24**	None detected	Undetermined
**CP 27**	None detected	Undetermined
**CP 49**	None detected	Undetermined
**CP 51**	None detected	Undetermined
**CP 102**	None detected	Undetermined
**CP 109**	None detected	Undetermined
**CP 122**	None detected	Undetermined
**CP 123**	None detected	Undetermined

**Figure 3:**
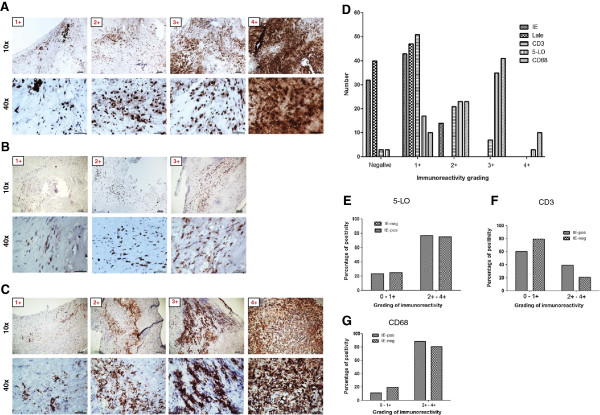
**Immunoreactivity and grading of inflammatory markers in association to human cytomegalovirus (HCMV) antigens burden. (A-C)** Immunoreactivity and grading of 5-lipoxygenase (5-LO) **(A)**, CD3 **(B)** and CD68 **(C)** in plaques. **(D)** Summary of grading results for HCMV immediate early (IE), late antigen (LA), 5-LO, CD3 and CD68 in plaques. **(E-G)** Association of HCMV IE antigens with 5-LO **(E)**, CD3 **(F)** and CD68 **(G)**. Positivity was revealed by diaminobenzidine (DAB), brown products.

To quantify HCMV immunoreactivity in CEA samples, we graded positive staining (Figure 2F-2G) as previously described [29]. HCMV immunoreactivity was either grade I or grade II. Grade I was the most prevalent for IE (43/57 or 75%) and all LA positive samples were grade I (Figure 3D). Cells expressing HCMV antigens were mainly detected in the shoulder regions of the plaques, in areas adjacent to the necrotic core and occasionally also in the fibrous cap (Figure 2F, 2+). The cellular targets for HCMV infection was mainly macrophages/foam cells and smooth muscle cells although we also detected viral antigens in some endothelial cells and other infiltrating inflammatory cells (Figure 2B-2D and Figure 2F-2G).

To address the question whether any association exists between HCMV IgG titers and viral burden in carotid plaques, we combined the results obtained from the serology and viral IE antigens staining analyses. Among 51 patients who were positive for HCMV IE, 10 (20%) patients had O.D. < 1 and 41 (80%) patients had O.D. > 1. Among 30 HCMV IE negative patients, 6 (20%) had O.D. < 1 and 25 (80%) had O.D. > 1 (Figure 2H). Thus, we did not observe any association between viral antigen burden in the plaque and HCMV IgG levels.

### HCMV trended to be associated with increased inflammatory burden

We next assessed if there was an association between HCMV infection and inflammatory markers. We stained tissue samples from CEA patients for 5-LO, CD3 and CD68; -all representing markers of inflammation. We found that 78/81 (96%), 79/82 (96%) and 84/84 (100%) of the CEA plaques were positive for 5-LO, CD3 and CD68, respectively (Figure 2A). We also graded these samples according to positivity (grade I-IV) as exemplified in Figure 3A-3C and summarized in Figure 3D. 5-LO proteins were abundantly expressed in macrophage/foam cells and in smooth muscle cells; 38 of 81 (47%) samples were grade III or IV (Figure 3D). All examined plaques were positive for CD68 (Figure 3D); 51 of 84 (61%) samples were grade III or IV (Figure 3D). CD3-positive cells were less abundant; 51 of 82 (62%) samples were grade I, none was grade II-IV. Thus, CD68-positive macrophages and 5-LO immunoreactivity were highly abundant in atherosclerotic plaques, relative to CD3-positive T cells that were less frequently observed in CEA plaques (Figure 3D).

Next, we divided samples by the grade of HCMV IE expression and compared staining results from 5-LO, CD3 and CD68 to determine whether IE positivity was associated with a higher grade (grade II and above) of the inflammatory markers. We found no statistically significant difference in the grade of inflammatory markers between those that were low HCMV IE vs. high HCMV IE reactivity: IE vs. 5-LO (p = 1.0000); IE vs. CD68 (p = 0.3408) and IE vs. CD3 (p = 0.0920). However, we observed a trend for higher immunoreactivity among HCMV-positive plaques for CD3 and CD68 compared to that of HCMV-negative plaques (Figure 3E-3G), indicating that HCMV may be involved in driving the inflammatory process or may be associated with this process.

## Discussion

We found a high prevalence of HCMV protein expression in atherosclerotic plaques, suggesting an ongoing active HCMV replication in a majority of atherosclerotic plaques. As viral antigens by their nature as foreign antigens provide targets for the immune system, it is plausible that some immune reactivity existing in the plaque is directed against HCMV peptides. This statement is supported by the fact that we found a trend for increased number macrophages and T cells in HCMV-positive plaques.

The life cycle of HCMV intrinsically involves macrophages and inflammation. HCMV maintains latency in myeloid lineage cells and becomes reactivated in macrophages differentiated by inflammatory stimuli [31]. HCMV can induce inflammation by inducing 5-LO and COX-2 expression with consequent leukotriene and prostaglandin E2 production [25,32]. Furthermore, HCMV also increases the production of pro-inflammatory cytokines, *e.g.* IL-1β, IL-4, IL-6, IL-8, TNF-α and IFN-γ [33]. The HCMV US28 and IE proteins induce production of IL-6 [32,34], which provides a direct link to C-reactive protein (CRP) levels. Both IL-6 and CRP are widely used markers for systemic inflammation and predictors of coronary heart disease [35].

We detected HCMV IE DNA in 44% (n = 9) of the HCMV antigen positive plaques while no HCMV IE DNA existed in HCMV antigen negative plaques (n = 9). In 75 arterial vascular tissues, a similar detection rate of HCMV DNA was reported [36]. As we found HCMV present mainly in the shoulder region and we had access to DNA from undefined parts of the plaques, it is possible that viral DNA was absent in some of the samples analyzed. Our PCR assay has a sensitivity of 765 IU/ml, which is also a limiting factor. Furthermore, it is possible that some clinical HCMV strains are not detected by our assay, which is supported by our own unpublished data from cancer patients who demonstrate variability in the IE and gB genes sometimes resulting in lack of detection of viral genes by both our PCR assays. We also failed to retrieve RNA in sufficient quantity and quality from 10 samples to proceed with cDNA synthesis or PCR analysis. It is possible that failure to retrieve RNA was due to low amount of RNA in the necrotic core of the end-stage plaques and we were unable to determine exactly from where in the specimen the section came from. Also it is well-known that RNA is sensitive to degradation depending on storage conditions, which may also explain why we did not recover enough RNA for further analyses.

To our knowledge, only two earlier reports examined the presence of HCMV protein expression in atherosclerotic plaques with reasonably large sample cohorts [37,38]. Chiu et al. reported that 27/76 atherosclerotic plaques obtained from Canadians (35.5%) were immunoreactive for HCMV early antigens [37], and Yi et al. detected a similar HCMV IE positivity (12/35 or 34.2%) and less expression of HCMV LA antigens (4/35 or 11.4%) in samples from Chinese Han patients [38]. We detected HCMV IE antigens in 57/89 (64%), and LA antigens in 47/87 (54%) of atherosclerotic plaques obtained from Russian patients. The higher percentage of immunoreactivity against viral antigens observed in this cohort may be due to different sample handling; both Chiu et al. and Yi et al. examined formalin-fixed samples, while we used frozen samples. Detection of HCMV proteins in cancer specimens is facilitated by antigen retrieval protocols, which results in enhanced sensitivity of the assay to similar detection levels as is observed in frozen tissue sections [39]. The HCMV serostatus of 99% (89/90) was also higher in our cohort than reported by Chiu et al. (50/76 or 65.8%) while not examined by Yi et al. Thus, ethnic differences may exist [40] regarding the prevalence of HCMV in CEA patients.

Elevated antibody levels against HCMV was shown by others to correlate positively with atherosclerosis [12], coronary artery disease [13] and CVD mortality [5,14-16]; but a lack of correlation between HCMV antibodies in patients with CEA compared with controls, were also reported [37,41]. In the present study, we did not observe a significant difference between the prevalence of HCMV IgG antibodies in patients with CEA compared to controls; both groups had similar IgG prevalence. However, we did observe lower viral O.D. values in patients than controls. It is possible that the adaptive immune response to HCMV is less efficient in individuals who are at higher risk of developing atherosclerosis, or that the virus by itself provides immunosuppressive mechanisms. It is also well known that HCMV has developed multitude of sophisticated mechanisms to avoid detection by the immune system [31,42,43], which may also explain the high prevalence of HCMV antigen reactivity in CEA patients, who had lower OD values than controls.

The concept of a low grade infection is in line with emerging views that there is an association between chronic low grade inflammation or infection and the slow process of arteriosclerosis [44]. HCMV induces expression of both 5-LO mRNA and proteins in addition to LTB4 production in infected smooth muscle cells [25]. Here, we detected 5-LO expression in HCMV-infected smooth muscle cells besides macrophage/foams cells, which substantiate our previous findings that 5-LO expression can be induced in smooth muscle cells [25] that otherwise do not express 5-LO. We observed that the proportion of 5-LO positive plaques were higher in plaques with higher HCMV antigen burden, but was not higher in HCMV positive versus negative plaques. Thus, other mechanisms than HCMV can also induce 5-LO mediated inflammation in vascular cells. High number of CD68-positive cells were associated with 5-LO expression levels (Figure 3B-3C), indicating that the main source of 5-LO was from macrophages, as was previously proposed [45]. Carotid atherosclerotic plaques containing enhanced numbers of CD3- and CD68-positive cells as indicators of increased inflammatory activity trended to be more often HCMV positive.

In summary, HCMV IE and LA antigens are abundantly expressed in carotid atherosclerotic plaques obtained from Russian patients. The seroprevalence of HCMV was very high in both Russian patients with CEA as well as in controls, but the IgG titers were significantly lower among patients than controls. There was abundant 5-LO expression and a substantial infiltration of CD68-positive macrophages in plaques, but relatively less prevalent infiltration of CD3-positive T cells. We observed a trend for increased infiltration of CD3-positive T cells and macrophages in HCMV-positive plaques, suggesting that this virus may influence the inflammatory activity in atherosclerotic plaques.

## Abbreviations

HCMV: Human cytomegalovirus; IE: Immediate early proteins; TBS: Tris-buffered saline; CEA: Carotid endartectomy; DAB: 3, 3-diaminobenzidine; SD: Standard deviation; 5-LO: 5-lipoxygenase; SPICE: St. Petersburg Investigation of Carotid Endarterectomies; CVD: Cardiovascular disease; O.D.: Optical density.

## Competing interests

The authors declare that they have no competing interests.

## Authors’ contributions

KCY and CSN conceived and designed the study. KCY, CT, AAM and BD performed all the experiments, analyses and generated figures. OO, ES, OR and AK provided materials crucial for the study. KCY wrote the manuscript. CSN, VW, AR, LB and AA participated in the analyses and helped in drafting the manuscript. All authors have read and approved the final manuscript.
